# A Time-Varying Filter for Doppler Compensation Applied to Underwater Acoustic OFDM

**DOI:** 10.3390/s19010105

**Published:** 2018-12-29

**Authors:** Habib Mirhedayati Roudsari, Jean-François Bousquet

**Affiliations:** Department of Electrical and Computer Engineering, Dalhousie University, Halifax, NS B3H 4R2, Canada; mirhedayati@gmail.com

**Keywords:** acoustic communication, wideband channel, channel modelling, time-scaling, doppler compensation, multicarrier modulation

## Abstract

This paper describes a Doppler compensation algorithm to improve the reliability of orthogonal frequency division multiplexing (OFDM). To compensate for the time-varying Doppler effect in a mobile deployment scenario, first the time-scaling factor over a wideband channel is estimated using pilot tones inserted in each OFDM symbol. Then, using a time-varying resampling technique, the Doppler effect is compensated during the reception of each OFDM symbol in the frame. To predict the performance of the system in relatively shallow waters, a software channel model is developed that is able to simulate a wide variety of dynamic shallow water deployment scenario. The performance of the algorithm was tested for two extreme frequency ranges during sea trials, the first at 2 kHz for a long-range application, and the second at 125 kHz for a short range telemetry link. For the 2-kHz system, a 16-bps mobile link in which the platform was moving at 1 m/s was demonstrated to have a bit error rate on the order of $10^{-3}$, while, for the 125-kHz telemetry application, a 2000-bps link was enabled with a bit error rate of 0.03 at a low SNR equal to 5.5 dB.

## 1. Introduction

The development of underwater communication systems is being spearheaded by the investment of infrastructure including autonomous underwater vehicles (AUVs) along the coasts for commercial, environmental and military applications. Shallow water deployments are of particular interest because of the increased industrial activity in these regions. However, underwater wireless data transmission suffers from significant channel variations.

To enable a spectrally efficient communication link underwater, orthogonal frequency division multiplexing (OFDM) is a multi-carrier transmission scheme that has received increased interest in wireless communications due to its low complexity channel estimation and equalization. The key feature that defines OFDM is that the sub-channels are separated by the minimum frequency separation while maintaining orthogonality. The orthogonality allows simultaneous transmissions on adjacent sub-channels to not interfere with each other. For underwater acoustic communications, OFDM has been proposed in different works [1,2,3], since it promises much greater data rates than single carrier modulation based schemes [4], and especially in comparison to non-coherent frequency shift keying techniques [5]. However, it is well recognized that OFDM is subject to time-variance. As such, the objective of this paper is to model and assess the performance of underwater acoustic communication systems relying on multi-carrier transmission subject to high mobility.

Most wireless communication systems rely on a narrowband representation of the signal where the bandwidth is much smaller than the carrier frequency. Then, temporal variations due to mobility in the channel can be simply represented using a linear fading process on each tap arrival. However, as explained in [6], in wideband communication systems, mobility must be characterized by time/frequency scaling. In addition, in a multipath environment, the angle of arrival and departure are typically different for each path arrival, and as such induce different Doppler shifts in each path. This generates a frequency dispersion of the transmit signal [7].

In this paper, a low-complexity filter originally introduced in [8] is utilized to model instantaneous channel variations due to vessel velocity and acceleration. The proposed technique can be used to enhance currently existing channel models, including geometry based simulators relying on ray tracing for example [9], or statistical models described in [7]. The methodology proposed allows compression and dilation of the signal as a function of time since it implements a time-variant convolution, and the filter utilized re-samples the signal, in a similar fashion as those used in delay locked loops.

It is generally accepted that temporal variations of the channel are adverse to the coherent reception of OFDM, because the energy of the subcarriers spills over into adjacent bands. The effect is even more difficult to address in mobile conditions because the coherence time is short and the channel signature may even change within the transmission of long OFDM symbols. A few techniques have been described in the literature to mitigate Doppler [1,2,10,11,12,13]. In [12], a methodology to estimate the carrier frequency offset (CFO) on the individual subcarriers is proposed. However, this technique relies on accurate channel state information. In [1], a two-step approach is taken, in which a coarse estimation of the time-scaling factor over the packet is accomplished using the preamble and postamble. Then, the residual frequency shift is estimated by minimizing the spillover energy in null subcarriers. Since a common time-rescaling factor is used for the entire duration of the frame, this technique is not optimal when there are sudden variations in equivalent speed between the two platforms. Similarly, in [13], the authors proposed a simple time-offset estimator that utilizes the cross-correlation of the cyclic prefix and its replica at the end of each OFDM symbol to estimate the Doppler scaling factor. In contrast to the work described in [1,13], this paper attempts to compensate the Doppler effect due to large velocity variations that occurs during the transmission of the frames.

For the techniques described in [1,12,13], the time-scaling factor is assumed to be common to all paths. In [14], the authors demonstrated the optimal time-scaling factor in a multipath channel in which each path is subject to a different time-scaling factor. The algorithm proposed in [14] is borrowed in this work and a wideband channel model is developed to represent realistic conditions. Note that, in [14], the authors assumed that the time-scaling factor over the period of a packet remains constant and the estimate of this factor is measured only at the start of each packet. Instead, as shown in this work, a new estimate of the time-scaling factor can be obtained using pilot tones in each OFDM symbol. Moreover, to maintain link reliability throughout the transmission of the frame, the time-scaling factor is interpolated on a sample by sample basis to further improve the Doppler shift compensation.

To demonstrate the algorithm accuracy, the receiver performance was evaluated in controlled conditions, as well as with realistic data taken for two extreme deployment scenarios: long range low-bit rate transmission, and short-range high-bit rate transmission. For the long-range application, Doppler compensation was demonstrated for a variation in relative speed between ±1 knots. The probability of error obtained after Doppler compensation was on the order of $10^{-2}$, for a data transmission rate of 16 bps, and a code rate of 1/16. To further improve the performance, multiple receiver elements can be utilized. As shown below, the Doppler compensation allows recovering a similar bit error rate as that obtained when the platform is fixed. In addition, for a short-range telemetry application with mobile nodes, a design in the 125 kHz frequency range was deployed to establish a 2 kbps link, and the performance measured was shown to be very close to the performance obtained using a model of the environment. As shown below, for Config. #2, the measured bit error rate at a 5.5-dB was equal to 0.035, for a coded bit rate of 776 bps while the vessel was moving at 0.9 m/s over a distance on the order of 100 m. Simulations also predicted that the reliability asymptotically improves with SNR, and a bit error rate on the order of $10^{-3}$ could be achieved.

The paper is organized as follows. In Section 2, a mobile acoustic propagation model is proposed. In Section 3, the receiver implementation to mitigate the effect of Doppler shift is proposed. In Section 4, application to two extreme scenarios measured in real conditions are presented. Finally, in Section 5, the work is summarized.

## 2. A Low-Complexity Digital Filter to Model Mobility

In this section, a channel propagation model is enhanced to account for mobility. A relatively simple model is developed that creates dilation of the signal as it propagates along different paths, and as a function of the relative speed between the two nodes. A shallow water environment is assumed in which the water depth is much smaller than the distance between the nodes. This covers a wide range of challenging applications, e.g. in harbours, on the littoral, and long-range transmission in the Arctic. First, in Section 2.1, the physical effect of mobility on acoustic propagation is described. Then, in Section 2.2, a low-complexity time-variant filter is applied to accurately represent time-variant mobile conditions during data transmission.

### 2.1. Acoustic Propagation in Mobile Conditions

In this section, the effect of mobility on the propagation model is quantified as a function of the node speed relative to the propagation speed. The focus is primarily on platform mobility in the network, and can also loosely be extended to the mobility of scatterers including waves at the surface.

In an acoustic propagation channel, the channel impulse response, h(t,τ), is a function of time *t* and delay τ. It is expressed as a sum of $N_{p}$ time delayed and amplitude scaled versions of the transmitted impulse function [6], such that

(1) $$h\left(t,\tau \right)=\sum_{p=1}^{N_{p}}h_{p}\left(t\right)\delta \left(\tau -\tau_{p}\left(t\right)\right),$$

where, for path *p*, the amplitude $h_{p}\left(t\right)$ and the delay $\tau_{p}\left(t\right)$ vary as a function of time. For a propagation distance $r_{p}\left(t\right)$ traveled by the *p*th ray, the path delay $\tau_{p}\left(t\right)$ is

(2) $$\tau_{p}\left(t\right)=\frac{r_{p}\left(t\right)}{c_{w}},$$

where $c_{w}$ is the speed of sound in water.

Next, a simple geometrical model of the environment is proposed to obtain the path delay $\tau_{p}\left(t\right)$ as a function of time. Let us assume a pair of platforms moving in a direction parallel to the surface, such that the relative velocity $\vec{v(t)}$ between a transmitter moving at $\vec{v_{tx}\left(t\right)}$ and receiver moving at $\vec{v_{rx}\left(t\right)}$ is $\vec{v(t)}=\vec{v_{tx}\left(t\right)}-\vec{v_{rx}\left(t\right)}$. Then, the time-varying distance between the transmitter and receiver measured at the surface is $d\left(t\right)=\int_{0}^{t}\vec{v(t)}dt+d_{0}$ where $d_{0}$ is the initial distance between the two nodes.

**Figure 1:**
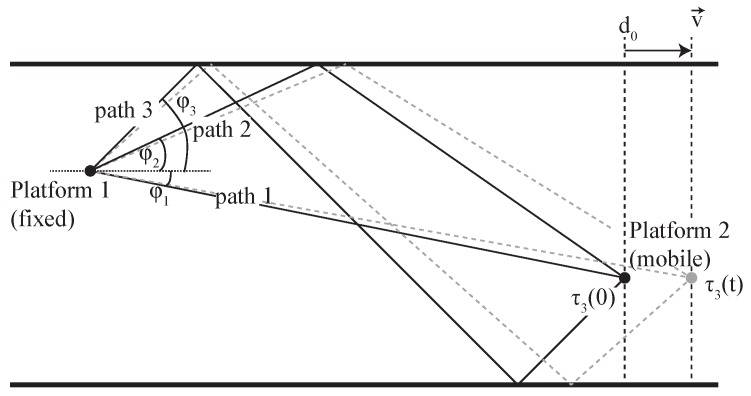
Representation of the multipath propagation behaviour in a shallow water mobile deployment.

In shallow water environments such as shown in Figure 1, the propagation delay of each ray after several bounces from the bottom and surface can be calculated geometrically as demonstrated in [15]. Using this model, the total propagation distance $r_{p}\left(t\right)$ is evaluated using simple trigonometry. The model can also be extended to a 3D one to consider reflections from shore lines, or other reflectors that may enclose the channel. However, the 3D model is out of the scope of this paper.

Referring to Figure 1, let us define at a mobile receiver platform an angle of arrival $\phi_{p}\left(t\right)$ with respect to the sea surface for the *p*th path arrival. The platform receives at time t=0 a path arrival from a transmitter at distance $d_{0}$, and that at this initial time the propagation delay is $\tau_{p}\left(0\right)$. Then, at time *t*, the propagation delay $\tau_{p}\left(t\right)$ can be roughly estimated as

(3) $$\tau_{p}\left(t\right)=\tau_{p}\left(0\right)+\frac{1}{\cos(\phi_{p}\left(t\right))}\left(\frac{v(t)}{c_{w}}t\right),$$

where $v\left(t\right)=|\vec{v(t)}|$ is the velocity between the transmitter and receiver, in the direction of propagation. This expression indicates that the different path delays vary somewhat differently because of the different angles of arrival $\phi_{p}\left(t\right)$ on each path. Then, the time-scaling factor for the *p*th path $\alpha_{p}\left(t\right)$ describes the rate of the received signal compression or dilation. It is defined as

(4) $$\alpha_{p}\left(t\right)=\left(1+\mu_{p}\left(t\right)\right)=\left(1+\frac{v_{p}\left(t\right)}{c_{w}}\right),$$

where $v_{p}\left(t\right)=v/\cos(\phi_{p}\left(t\right))$ and $\mu_{p}=v_{p}/c_{w}$ is defined as the Mach factor. When $v_{p}\left(t\right)<0$ and $\alpha_{p}\left(t\right)<1$, the path length is decreasing, which causes compression in the time domain. Similarly, the signal would be dilated in time domain when $v_{p}\left(t\right)>0$ and $\alpha_{p}\left(t\right)>1$. Then, the delay as a function of the time-scaling factor $\alpha_{p}\left(t\right)$ for path *p* is

(5) $$\begin{array}{c} \tau_{p}\left(t\right)=\tau_{0,p}-\left(1-\alpha_{p}\left(t\right)\right)t. \end{array}$$

where $\tau_{0,p}$ is the *p*th path delay at the reference time t=0. Note that, in this model, only the motion of the vessel tangential to the sea surface is modelled; however, it would be possible to extend this model to also account for the vertical motion of the node. Then, the time-varying channel impulse response expressed in Equation (1) becomes

(6) $$h\left(t,\tau \right)=\sum_{p}h_{p}\left(t\right)\delta \left(\tau -\tau_{0,p}+\left(1-\alpha_{p}\left(t\right)\right)t\right).$$

In this work, the acoustic propagation is modelled for relatively shallow environments, in which the sound velocity is assumed to be constant and equal to $c_{w}=1500\;m/s$. In reality, the sound speed profile can vary as a function of depth, and this can induce different Doppler scaling factors for each path. In this scenario, the proposed model must be augmented, since this would induce different time-scaling factors $\alpha_{p}\left(t\right)$ for each path *p*.

It is also useful to represent the channel frequency spectrum, particularly for multicarrier transmission. In [7], the channel frequency response at time instant *t* is obtained by taking the Fourier transform of Equation (6) with respect to τ such that its frequency response as a function of time *t* and frequency *f* is

(7) $$H\left(t,f\right)=\sum_{p}h_{p}\left(t\right)e^{-j2\pi f\tau_{0,p}}e^{j2\pi \frac{v_{p}}{c_{w}}ft},$$

where the first exponential term $e^{-j2\pi f\tau_{0,p}}$ indicates that there is a frequency dependent phase shift of $-2\pi \tau_{0,p}f$ and the second term $e^{j2\pi \frac{v_{p}}{c_{w}}ft}$ shows a frequency dependent frequency shift of $\frac{v_{p}}{c_{w}}f$ on the *p*th path due to the relative speed $v_{p}$. When the bandwidth *B* of the signal is relatively small with respect to the carrier $f_{c}$, the phase shift at time *t*, $2\pi \frac{v_{p}}{c_{w}}ft$ can be assumed constant over all the frequencies within the bandwidth. However, if the narrowband condition is not respected, then the Doppler effect cannot be modelled as a constant frequency shift for all frequencies [1]. In this case, it is preferable to model the Doppler effect using a filter that allows time-variant time-scaling, as described in the next section.

### 2.2. Time Varying Fractional Delay Line

In this section, a filter that relies on a fractional delay line is proposed to model the channel time-variance. In [8], a similar filter is proposed, and is commonly used in delay locked loops. In this work, a low complexity implementation is applied, in a first instance, to model mobility in the channel, and, in a second instance, to realize Doppler compensation.

Time-scaling is usually implemented by interpolating the data. Let us assume a signal x(t) sampled at a frequency $F_{s}$. Then, if x(t) is scaled by a constant factor, α, it can be resampled at frequency

(8) $$F_{interp}=\frac{1}{\alpha }F_{s}.$$

In other words, time-scaling is implemented by interpolating the signal at a frequency $F_{interp}$. The interpolated discrete signal is discretized at the original sample rate $F_{s}$ to model time-scaling. The time-scaling factor can be approximated as a rational number, M/N, that is typically very close to unity (for a relative speed *v* much smaller than the speed of propagation). Then, resampling can be implemented by: (1) up-sampling and interpolating at the rate *M*; and (2) down-sampling (decimating) the up-sampled signal at rate *N*. When the time-scaling factor is close to 1, *M* and *N* will be large and the resampling process is computationally expensive, which makes it inappropriate in hardware implementation when the memory is limited.

In this work, a *Time-Varying Fractional Delay Line* (TVFDL) is introduced as an alternative technique to realize time-variant resampling [16]. Using this method, the time scaling is implemented by sampling the original signal with a time-varying delay τ[n] at discrete time *n*. Using this approach, once the time-varying delay is calculated using Equation (2), it can be implemented using a dynamic resampling process.

To describe the TVFDL mathematically, assume x(αt) is a continuous-time time-scaled signal. The signal $x_{s}{\left[n\right]=x\left(\alpha t\right)|}_{t=nT_{s}}$ is the discrete-time representation with a sampling period of $T_{s}$ and for which *n* is a positive integer between 0 and $N_{max}-1$. Then, the signal $x_{s}\left[n\right]$ is

(9) $$\begin{array}{cc} x_{s}{\left[n\right]=x\left(\alpha t\right)|}_{t=nT_{s}} & =x(\left(1-\left(1-\alpha \right)\right)nT_{s})\\ & =x(nT_{s}-\left(1-\alpha \right)nT_{s}). \end{array}$$

**Figure 2:**
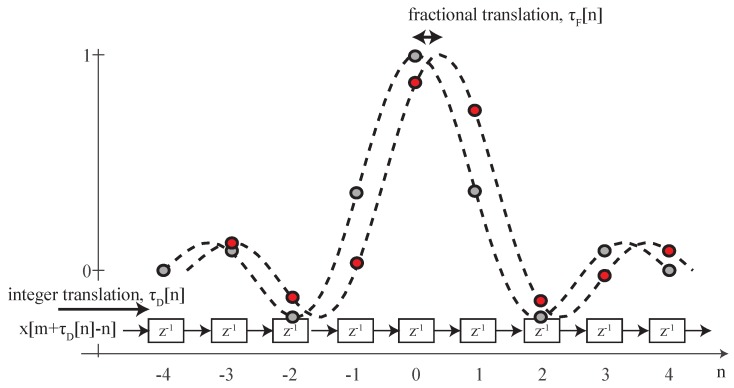
The time-varying fractional delay line: the signal is delayed by the integer value, while the sinc function implements the fractional delay.

The time-scaled signal can also be written as $x_{s}\left[n\right]=x\left(nT_{s}-\tau \left[n\right]\right)$, where $\tau \left[n\right]=\left(1-\alpha \right)nT_{s}$. The interpolation filter is a sinc function and is implemented by a tapped delay line, as shown in Figure 2. The sinc filter bandwidth $B_{f}$ is defined to be equal to that of the signal bandwidth, i.e., $B_{f}=1/T_{s}$. As such, the time delayed signal is

(10) $$x\left(nT_{s}-\tau_{s}\right)=x\left[n-\tau_{s}\right]=\sum_{m=-\infty }^{\infty }\mathrm{sinc}\left[B_{f}\cdot \left(m-\tau_{s}\right)\right]x\left[n-m\right],$$

where $\tau_{s}$ is the normalized delay with respect to the sampling period, $T_{s}$. The normalized delay $\tau_{s}$ can be broken into the integer part, $D_{\tau_{s}}$, and the fractional part, $d_{\tau_{s}}$, such that $\tau_{s}$ is

(11) $$\tau_{s}=\frac{\tau }{T_{s}}=D_{\tau_{s}}+d_{\tau_{s}}.$$

The TVFDL is realized by implementing an FIR filter that approximates the sinc function. To reduce its order to a finite length, the sinc filter is truncated. The order of the filter limits the filter accuracy and the length is a trade-off between the precision of the estimated signal value at the delay of interest and the computational complexity associated with this estimation. If the length of the FIR filter is chosen as 2M+1, then higher values of *M* will lead to a longer computational time, but more accurate results [17].

As shown in Figure 2, in discrete-time, the total delay τ[n] at time *n* is implemented by an integer and a fractional part, such that $\tau \left[n\right]=\tau_{D}\left[n\right]+\tau_{F}\left[n\right]$. The integer part $\tau_{D}\left[n\right]$ indicates the number of taps by which the input signal will be delayed, and the filter coefficients take into account the fractional part $\tau_{F}\left[n\right]$ at a given time instant *n*. The position of this delay can then be updated for each time instant in order to implement the desired time-varying resampling.

Working with the fractional delay has two main benefits to model mobility. First, any resampling rate can be implemented with low computational complexity since the time-varying delay will simply be different for different resampling rates. Second, this model accounts for dynamically changing velocity between the receiver and transmitter, because it relies on the time-varying delay, $\tau_{p}\left(t\right)$, which can be simply calculated for any dynamic scenario.

**Figure 3:**
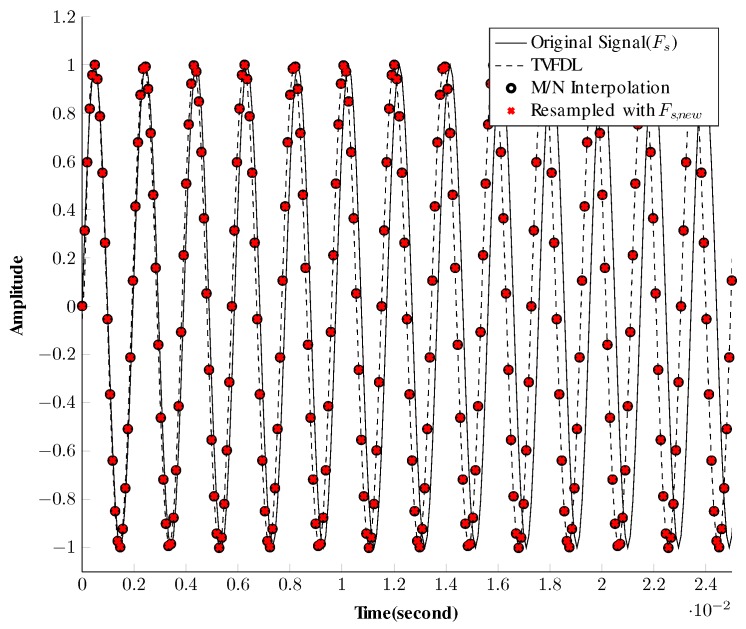
Comparison between different resampling methods applied to a 512-Hz sine wave with a sampling frequency of $F_{s}$ = 10,240 Hz.

To verify the functionality of the TVFDL, the resampling operation is applied to a reference waveform using different resampling methods and the results are compared in Figure 3. In this simulation, the scaling factor α=1.0167, which is equivalent to a constant relative velocity of $|v_{c}|=+25\;m/s$, and compresses the received signal. The sound velocity $c_{w}$ is expected to be constant along the water column and is equal to 1500 m/s. As shown in Figure 3, the TVFDL has a similar result to linear interpolation methods.

To demonstrate the ability of TVFDL in implementing a wide variety of mobile deployment scenarios, a deterministic channel model with four paths is implemented. As described in [16], the time-varying characteristics associated with each path are modelled using different geometric functions. Specifically, the first and second paths have a constant velocity of v=0 and v=2m/s, respectively. In addition, the third and fourth paths are subject to a time-varying velocity with a dynamic and constant acceleration rate, respectively. Note that, for demonstration purposes, the amplitude of these paths is constant. While the objective here is to demonstrate the flexibility of the TVFDL, physical channel models developed using the proposed technique are validated against real measurement results in [16].

**Figure 4:**
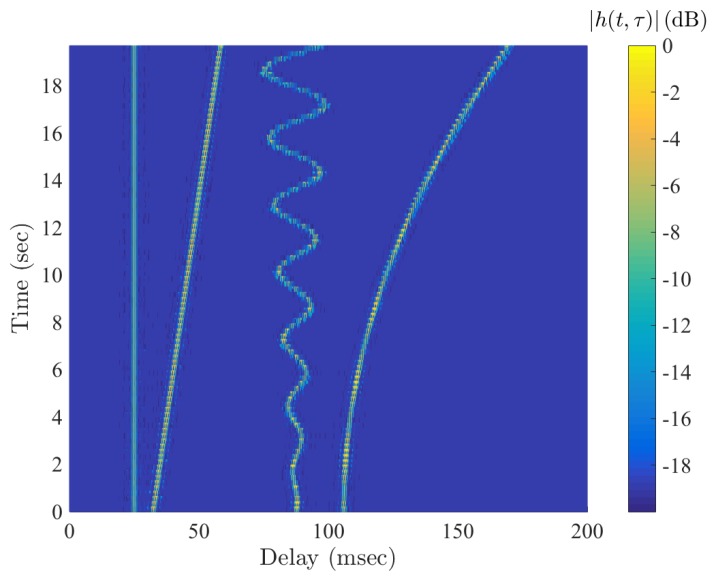
Examples of implementation of time-varying delay using TVFDL.

To verify the functionality of this model, the channel impulse response is estimated using a cross-correlation filter and the impulse response as a function of time is shown in Figure 4. The output demonstrates the capacity of the model to represent various mobility conditions. In the next section, a receiver is described to compensate for Doppler shift.

## 3. Doppler Compensation for Multicarrier Modulation

In this section, an algorithm to compensate for Doppler is described. In comparison to the algorithm described in [18], a common Doppler compensation is applied to reduce computational complexity. First, in Section 3.1, a mathematical model is developed to represent the effect of time-variance on the received signal, and then, in Section 3.2, signal processing techniques at the receiver to compensate time-scaling are developed.

### 3.1. Impact of Mobility on Signal Integrity

In a wideband communication channel, time-scaling due to time-variance introduces a frequency-dependent frequency shift. Because of this, during the transmission of a multicarrier modulated signal, the energy of a sub-carrier spills over to adjacent sub-carriers. This phenomenon is called *inter-carrier interference* (*ICI*). In this section, the analytical model used to represent the signal at the receiver is provided. The model is similar to that presented in [14], but uses as its input the relative speed and acceleration of the platforms. In addition, in this work, pilot tones inserted in each OFDM symbol serve to refine the Doppler estimation even at high mobility.

For a time-scaling factor that is dependent on platform speed, the time-varying channel impulse response defined by Equation (1) can be expressed as

(12) $$h\left(t,\tau \right)=\sum_{p=0}^{P-1}h_{p}\delta \left(\tau -\tau_{p,0}+\frac{1}{c_{w}}\left(v_{p,0}+\frac{a_{p}}{2}t\right)t\right),$$

where $v_{p,0}$ is the initial velocity, $a_{p}$ is the average acceleration of the *p*th path and $h_{p}$ is assumed to be constant as a function of time.

To analyze the effect of a constant time-scaling on an OFDM symbol, the transmitted OFDM symbol is rewritten as a sum of a number of orthogonal complex sinusoidal functions, such that

(13) $$s_{m}\left(t\right)=\sum_{k=-N/2}^{N/2-1}s_{k}e^{j2\pi f_{k}t}u\left(t\right),$$

where $s_{k}$ is the *k*th data symbol and u(t) is a rectangular pulse shaping function, expressed as

(14) $$u\left(t\right)=\left\{\begin{array}{cc} \sqrt{\frac{1}{T}} & \mathrm{if}\;t\in [0,T].\\ 0, & \mathrm{otherwise}. \end{array}\right.$$

The *k*th sub-carrier frequency is $f_{k}=f_{c}+k\Delta f$, where Δf is the frequency separation between the sub-carriers and $k=-\frac{N}{2},\cdots ,\frac{N}{2}-1$. The *m*th received OFDM symbol can be expressed as [14]

(15) $$\begin{array}{ll} r_{m}\left(t\right) & =\sum_{p=0}^{P-1}h_{p}s_{m}\left(\left(\mu_{p,m}+1\right)t-\tau_{p,0}\right)+n\left(t\right)\\ & =\sum_{p=0}^{P-1}h_{p}\sum_{k=-N/2}^{N/2-1}s_{k}e^{j2\pi f_{k}\left(\left(\mu_{p,m}+1\right)t-\tau_{p,0}\right)}u\left(t\right)+n\left(t\right)\\ & =\sum_{p=0}^{P-1}h_{p}\sum_{k=-N/2}^{N/2-1}s_{k}e^{j2\pi f_{k}t}e^{-j2\pi f_{k}\tau_{p,0}}e^{j2\pi (f_{k}\mu_{p,m})t}u\left(t\right)+n\left(t\right). \end{array}$$

where $\mu_{p,m}$ is the Mach factor at path delay *p* at OFDM symbol *m*. At the receiver, the baseband signal is obtained by down-converting and then sampling $r_{m}\left(t\right)$ at T/N seconds [14]. It is described in discrete-time by

(16) $$\begin{array}{ll} r_{m}\left[n\right] & =r_{m}\left(t\right)e^{j2\pi f_{c}t}{|}_{t=nT/N}\\ & =\sum_{p=0}^{P-1}h_{p}\sum_{k=-N/2}^{N/2-1}\left(s_{k}e^{-j2\pi f_{k}\tau_{p,0}}\cdot e^{j2\pi k\left(1+\mu_{p,m}\right)\frac{n}{N}}\cdot e^{-j2\pi f_{c}\mu_{p,m}n\frac{T}{N}}\right)+n\left[n\right] \end{array}$$

where n=0,1,⋯,N−1. The discrete-time *m*th received OFDM symbol sampled at the data rate is represented in a vector form as [14]

(17) $$\begin{array}{cc} r_{m} & =[r(0),r(1),\cdots ,r(N-1)],\\ r_{m} & =\sum_{p}h_{p}D_{p,m}A_{p,m}\Gamma B_{p,m}s+n, \end{array}$$

where $D_{p,m}$ and $B_{p,m}$ are N×N diagonal matrices, which model, respectively, the frequency offset and phase shift in the *p*th path at the *m*th OFDM symbol. $A_{p,m}$ is an N×N Vandermonde matrix that models frequency distortion due to time-scaling and Γ is an N×N selection matrix that selects the active sub-carriers ($N_{a}$). The matrices are populated using

$$\begin{array}{ccc} D_{p,m}\left(n,n\right) & =e^{-j2\pi f_{c}\mu_{p,m}\left(nT/N\right)}, & n=0,1,\cdots ,N-1,\\ B_{p,m}\left(n,n\right) & =e^{-j2\pi f_{k}\tau_{p,0}}, & m\in N_{a},\\ \left[A_{p,m}\right]\left(n,k\right) & =e^{j2\pi nk(1+\mu_{p,m})/N}, & n,k=0,1,\cdots ,N-1, \end{array}$$

and

(18) $$\begin{array}{ccc} \Gamma_{n,k} & =\left\{\begin{array}{cc} 1, & \mathrm{if}\;n=k\;\mathrm{and}\;k\in N_{a},\\ 0, & \mathrm{otherwise} \end{array}\right. & n,k=0,1,\cdots ,N-1. \end{array}$$

The discrete-form vector representation of the received signal as expressed by Equation (17) is convenient because it can be directly modelled using digital signal processing simulation tools. It is used in the next section to provide an estimate of the optimal time-scaling factor.

### 3.2. Compensation at the Receiver

In this section, an algorithm to address time-variance and optimize link reliability at the receiver is proposed. First, a short discussion is offered on the optimum time-scaling factor in a mobile multipath environment, then an algorithm to rescale the received signal in real-time is described, and, finally, a full receiver architecture is provided to decode the data. Note that, while the Doppler scaling estimator is described in [14], the algorithm is modified to track the estimate between each OFDM symbol and interpolate the time-scaling on a sample-by-sample basis.

In mobile underwater conditions, the signal may arrive through path arrivals that are subject to different time-scaling factors. While it is often assumed that all paths have equal time-scaling factor, measurements have shown that, in a real environment, each path may experience a different time-scaling factor [19]. In this case, the optimal resampling factor is not clearly defined. Specifically, in [14], the authors analyze the effect of resampling using a single time-scaling factor on the performance of the receiver in a multi-scale multipath channel. They show that, when the spread $D_{s}$ of the channel time-scaling factor, called scaling spread, is large ($D_{s}>1\times 10^{-3}$), resampling with respect to a single scaling factor can cause degradation of reliability, because of the residual Doppler shift after resampling. In this case, the optimal time-scaling factor is equal to the time-scaling factor of the path which has the largest magnitude. In contrast, when the time-scaling factors are very close to each other, i.e., when the Doppler spread $D_{s}<10^{-3}$, it is shown that the optimum resampling factor is a weighted average of the time-scaling factors of all paths.

Earlier research on channel parameters estimation utilizes pilot sub-carriers to compensate for Doppler [12,14,20]. In most of these works, the Doppler shift is assumed to be constant during the packet and the estimation is performed using a block-type pilot arrangement. Using this methodology, it is not possible however to track a fast time-varying channel. Here, at the beginning of a given frame, a preamble is used to obtain a coarse estimation of the leading edge of the frame, and the following OFDM symbols have pilot tones as well as null sub-carriers strategically inserted between the payload carriers.

In [14], time-scaling is also realized for each OFDM symbol. However, the time-scaling compensation relies on a constant interpolation factor. Consequently, the receiver needs to re-synchronize between each OFDM symbol. Instead, in this work, the time-scaling factor estimation and OFDM block synchronization components at the receiver are interpolated to mitigate distortion due to time scaling on a sample-by-sample basis throughout the duration of the received frame.

To estimate the time-scaling factor, the received *m*th OFDM symbol can be expressed from Equation (16) using a single optimum resampling factor, α. The approximated received symbol is

(19) $$r_{m}\left[n\right]=\sum_{p=0}^{P}h_{p}e^{-j2\pi f_{k}\tau_{p,0}}\sum_{k=N/2}^{N/2-1}s_{k}e^{j2\pi k\left(1+\alpha \right)\frac{n}{N}}e^{j2\pi f_{c}\alpha n\frac{T}{N}}+n\left[n\right].$$

By separating the effect of multipath and Doppler, the approximated received vector is [14]

(20) $$\hat{r}=D_{\alpha }{\tilde{A}}_{\alpha }\Gamma x,$$

where $\hat{r}$ is an N×1 vector that represents the *N* samples of the received OFDM symbol. Note that x=Hs models the effect of multipath on the transmitted symbol, where H and s are the multipath channel matrix and transmitted signal vector, respectively. The matrix $D_{\alpha }$ models the frequency offset, and, in contrast to $D_{p,m}$ defined in Equation (17), the frequency offset is common to all paths since a single optimum time-scaling factor α is estimated. Similarly, ${\tilde{A}}_{\alpha }$ is a common frequency distortion due to time scaling for all paths. As previous defined in Equation (17), the selection matrix Γ identifies which sub-carriers contain pilot tones.

By using Equation (20), the mean squared error between the actual and the approximated received vector is defined as a cost function, which is an $L_{2}$ norm expressed as

(21) $$\varepsilon \left(x,\alpha \right)={∥r-\hat{r}∥}^{2}={∥r-D_{\alpha }{\tilde{A}}_{\alpha }x∥}^{2},$$

and is a function of the resampling factor α and of the multipath channel output x. When the channel parameters are known, the distorted reference x observed at the output of a static multipath channel is

(22) x=SFh,

where S is the diagonal $N_{p}\times N_{p}$ matrix of the pilot vector s, and $N_{p}$ is the length of the reference pilot vector. The matrix of F (with size $N_{p}\times P$) provides a relationship between the phase shift of each sub-carrier to the channel gain of each path and is given as

(23) $$F=\left[\begin{array}{cccc} e^{-j2\pi f_{p_{1}}\tau_{1}} & e^{-j2\pi f_{p_{1}}\tau_{2}} & \cdots & e^{-j2\pi f_{p_{1}}\tau_{P}}\\ e^{-j2\pi f_{p_{2}}\tau_{1}} & e^{-j2\pi f_{p_{2}}\tau_{2}} & \cdots & e^{-j2\pi f_{p_{2}}\tau_{P}}\\ \vdots & \ddots & \vdots \\ e^{-j2\pi f_{p_{P}}\tau_{1}} & e^{-j2\pi f_{p_{P}}\tau_{2}} & \cdots & e^{-j2\pi f_{p_{P}}\tau_{P}} \end{array}\right].$$

In addition, $h={\left[h_{1},h_{2},\cdots ,h_{L}\right]}^{T}$ is the L×1 vector of the channel gain on each path. An initial estimate of the channel impulse response, $\hat{h}$, can be obtained using the preamble as described in [1]. Then, the cost function in Equation (21) becomes

(24) $$\varepsilon \left(\hat{h},\alpha \right)={∥r-\hat{r}∥}^{2}={∥r-D_{\alpha }{\tilde{A}}_{\alpha }SF\hat{h}∥}^{2}.$$

In time-varying fading scenarios, the path amplitudes change from one symbol to the next. Thus, the coarse channel estimation from the preamble cannot be used for all the OFDM symbols. As explained in [14], since the statistical characteristics of the channel are unknown, the reference signal x can be estimated using

(25) $$\hat{x}={\tilde{A}}_{\alpha }^{†}D_{\alpha }^{H}r.$$

where ${\left(\cdot \right)}^{H}$ is the Hermitian adjunct operator, while ${\left(\cdot \right)}^{†}$ is the pseudo-inverse operator. Replacing $\hat{x}$ in Equation (25) with x in Equation (22), the approximation of the distorted received signal $\hat{x}$ can be expressed as

(26) $$\hat{x}={\tilde{A}}_{\alpha }^{†}D_{\alpha }^{H}r=SF\hat{h}.$$

Then, an estimation of the path amplitude, $\hat{h}$, can be obtained by applying a matrix inversion and multiplication on Equation (26), such that

(27) $$\hat{h}=F^{†}S^{H}{\left({\tilde{A}}_{\alpha }\right)}^{†}D_{\alpha }^{H}r.$$

The cost function then can be reduced to a one-dimensional function of the optimum resampling factor, α, by replacing Equations (27) in (24), such that the ML function becomes

(28) $$\varepsilon \left(\alpha \right)={\left|r-D_{\alpha }{\tilde{A}}_{\alpha }SFF^{†}S^{H}{\left({\tilde{A}}_{\alpha }\right)}^{†}D_{\alpha }^{H}r\right|}^{2}.$$

The optimum time-scaling factor is estimated by minimizing the new cost function as $\hat{\alpha }=\arg\;\min_{\alpha }\;\varepsilon$. It can be proven that the minimization problem is equivalent to maximizing the projection of the received signal onto the approximated received signal defined as

(29) $$\hat{\alpha }=\arg\;\max_{\alpha }\;r^{H}D_{\alpha }{\tilde{A}}_{\alpha }SFF^{†}S^{H}{\left({\tilde{A}}_{\alpha }\right)}^{†}D_{\alpha }^{H}r.$$

Note that the resolution of the estimate $\hat{\alpha }$ using Equation (29) increases with the number of pilot tones $N_{p}$, and also depends on the number of elements for the test vector α. As such, these parameters must be chosen carefully, because they also increase the computational complexity of the Doppler estimation algorithm. In addition, note that the time-scaling effect (compression/dilation) can move the start point of each OFDM symbol in the discrete-time domain. Therefore, a precise OFDM symbol synchronization is necessary before demodulation. It can be shown that, when the symbol synchronization is perfect, the projected energy in Equation (29) has the highest peak. As such, a technique to find each OFDM symbol’s start time can be combined with the optimum time-scaling estimation algorithm.

In summary, the procedure to find the time-scaling factor during reception of the frame is performed in three steps defined as follows.

1. Find a coarse estimate of the starting point of the *m*th OFDM block, $i_{0,m}$, by counting $m\times (N+L_{cp})$ samples after the preamble in the baseband.
2. To produce a fine estimate of each OFDM symbol’s start time, shift the sampling instant of the *m*th symbol around $i_{0,m}$ by an integer number of samples, *l*. Then, calculate for the ($i_{0,m}+l$)th delay the projection defined by Equation (29). The range of *l* depends on the intensity of Doppler and number of OFDM samples in discrete time-domain.
3. Choose the projection which gives the maximum peak between the energy functions as the optimum start point of the *m*th OFDM block.

Note that the resolution of the Doppler estimation algorithm is constrained on the size of the test sequence α to solve the ML algorithm, as defined by Equation (29).

**Figure 5:**
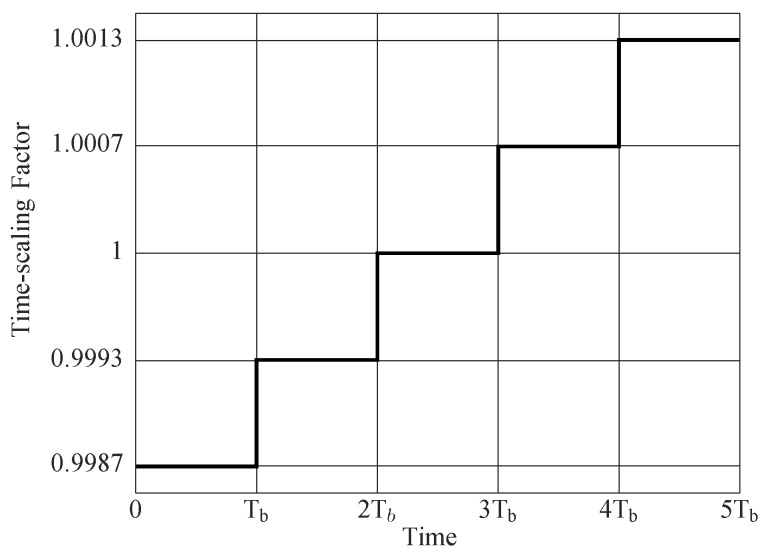
Step-like time-varying time-scaling function. The relative velocity during each OFDM block remains constant and it is changing from one block to the next. The vector of the relative velocity is $v_{r}=\left[-2,-1,0,1,2\right]$ m/s. The OFDM block duration is $T_{b}=1.9$ s.

Next, a simulation is presented for a packet of five OFDM symbols. The packet passes through a deterministic multi-scale multipath channel with a delay spread of $\bar{\tau }=59.3$ ms and a maximum scaling spread of $D_{s}=2.2\times 10^{-4}$. The signal to noise ratio is 10 dB. The time-scaling factor is constant during each OFDM symbol, and changes from one block to the next (see Figure 5). In this example, each OFDM symbol has 512 sub-carriers including 128 nulls and 128 pilots have been inserted in between them. The length of the cyclic prefix is 100 samples in baseband discrete-time. The transmitted signal bandwidth is 320 Hz. The range of integer sample search for symbol synchronization is l=[−4,4].

**Figure 6:**
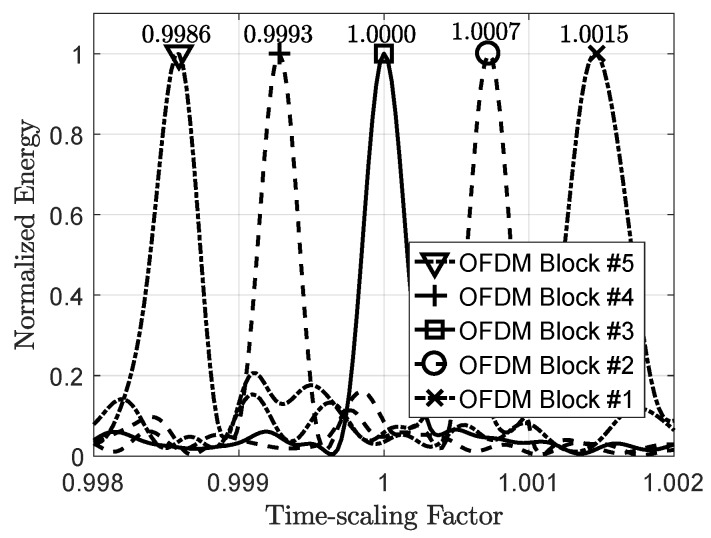
An example of linear search for time-scaling factor estimation in a packet of five OFDM blocks. The points for which the energies are maximized show the optimum time-scaling factors.

The maximum of the cost function for each OFDM symbol is obtained by a linear search on α. The result of the linear search is shown in Figure 6 where the estimation algorithm is able to track the time-scaling factors created by a relative speed up to 2.5m/s. Although this example demonstrates a constant speed for each block, the simulations showed that the estimation algorithm works in a continuously time-varying speed condition if the rate of acceleration remains small. The maximum acceleration depends on the length of each OFDM symbol and the central frequency. Basically, the change in velocity Δv during an OFDM symbol must be small enough so that the change in time-scaling factor Δα is much smaller than the estimated time-scaling factor, or, in other words, $\hat{\alpha }\gg \Delta \alpha$.

Since the time-scaling factor changes continuously in time, the received packet needs to be compensated with a time-varying resampling process. In this work, linear interpolation of estimates between the OFDM samples is adopted.

**Figure 7:**
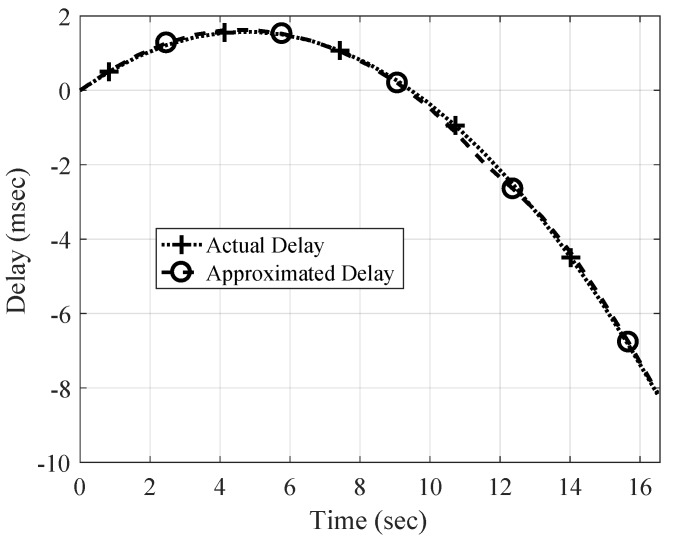
An example of time-varying delay estimation. The delay of the strongest path changes with time following a quadratic function.

Once the time-varying time-scaling factor over the packet is approximated by interpolation, the passband received signal can be resampled in a time-varying manner using the TVFDL. An example of the approximation of the time-varying delay is shown in Figure 7. In this channel simulation, the relative velocity between the receiver and transmitter is increasing continuously from −1 m/s to 2.5 m/s. The negative speed shows a positive delay and the positive speed creates a negative delay. The delay varies with time following a quadratic function because the acceleration rate remains constant for the packet duration.

The time-scaling estimator accuracy is limited. As shown in Figure 7, the linear interpolation can effectively approximate the time-varying time-scaling factor and consequently time-varying delay. However, this approximation has a residual error that can create considerable frequency-dependent frequency shifts after resampling. These residual frequency shifts can be modeled as a common frequency shift for all sub-carriers, if they are smaller than the frequency separation between the sub-carriers, Δf. This means that they can be canceled out by an opposite frequency shift. By this assumption, the maximum frequency shift is defined by

(30) $$\mu_{max}f_{max}=\left|\left(1-\alpha_{max}\right)\right|f_{max}<\Delta f,$$

where $f_{max}=f_{c}+\left(N/2-1\right)\Delta f$ is the maximum frequency component of the OFDM signal in passband. Then, the maximum tolerable Mach factor is

(31) $$\mu_{max}=\frac{\Delta f}{f_{c}+\left(\frac{N}{2}-1\right)\Delta f}.$$

This indicates that in a mobile UWA communication system design, choosing the optimum frequency separation and central frequency as well as the optimum sub-carrier allocation should be considered carefully. In the next section, the performance of the proposed Doppler compensation to track the time-scaling is presented for two realistic datasets.

## 4. Validation Using Real Data

Two major deployments for different applications were used to confirm the performance of multicarrier transmission in mobile conditions and to assess the performance of the Doppler compensation technique discussed in Section 3.2. In a first instance, in Section 4.1, an ultra-sonic telemetry link is presented and the reliability of the receiver deployed is compared to that of the software model for different system parameters. Then, in Section 4.2, a long-range communication deployment is described, and the receiver reliability is compared for different mobile conditions.

### 4.1. Short-Range Ultra-Sonic Transmission

In fall 2016, a sea trial was run in Shad Bay, Nova Scotia, to characterize the acoustic channel conditions in a shallow environment and to evaluate the performance of a short-range communication link, particularly for small telemetry nodes subject to mobility. The physical and geometrical parameters of the channel are summarized in Table 1. The transmitter consists of a digital signal processor interfaced to a wideband front-end operating at a center frequency near 125 kHz.

The transmitter was deployed at a depth of approximately 26.5 m. The transmit output power was controlled to maintain a signal-to-noise ratio approximately equal to 5.5 dB at the receiver. Pre-recorded sound files were sent using a 16-bit data acquisition card. Waveforms were defined to allow channel characterization and the assessment of a physical layer relying on OFDM.

At the receiver, the hydrophone projector was lowered to approximately 1.5 m from the sea surface. The receiver was attached to a boat, which was drifting at the surface with an average speed of approximately 0.3 m/s, as shown in Table 1. The received signal was saved to memory for post-processing.

**Figure 8:**
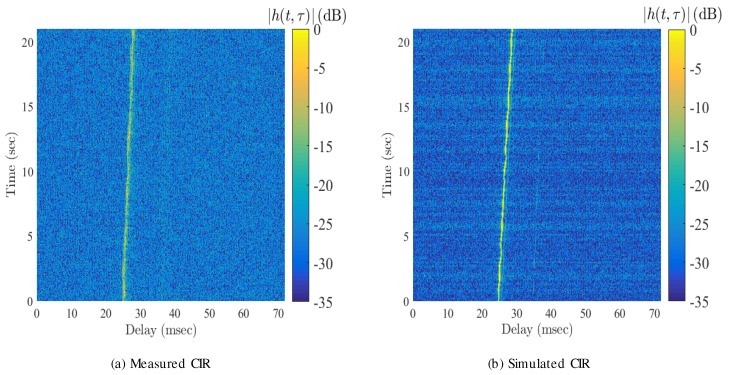
Measured and simulated channel impulse responses.

A standard cross-correlation channel sounder was implemented to extrapolate the channel impulse response as a function of time. Figure 8a shows the channel impulse response of the measured channel, while Figure 8b is the output of a shallow water channel simulator that was run for the defined deployment geometries. As can be observed, there is a strong first path arrival, and a second path can be faintly recognized at an additional delay of 5 ms. Since this deployment is in the ultra-sonic regime, it is expected that path delays that propagate over additional distance will suffer from much greater attenuation due to additional losses at high frequency, thus the main tap arrival is much stronger than for the later path arrivals. The channel amplitude remains relatively constant, and the main path delay increases from 26 to 30 ms within a 21 s time-span. The increasing path delay was corrected using the Doppler compensation algorithm described in Section 3. Note that, as can be seen, there is a good degree of resemblance between the measured and predicted model.

Next, to validate the Doppler compensation algorithm, each packet included a preamble for synchronization, followed by six OFDM symbols. There was a guard interval between the preamble and the OFDM symbols. A linear chirp signal was chosen as the preamble because of its robustness against Doppler.

To compare the performance of the Doppler compensation and equalization, two OFDM packet configurations were tested, and their parameters are summarized in Table 2. Different number of sub-carriers, number of pilot sub-carriers, bandwidth, central frequency and maximum tolerable frequency shift were chosen for the two configurations.

After frame synchronization, the received packets were decimated to the symbol rate. Then, the time-varying time-scaling factor estimator defined in Section 3 was applied to each packet. The estimated time-scaling factor for OFDM symbol was directly related to the relative velocity between the transmitter and receiver that was observed during this symbol.

After Doppler compensation and block synchronization, the channel impulse response on each OFDM block was estimated using the least-square (LS) method and the received block was equalized. Finally, the equalized signal was applied to the demodulation and decoding blocks. The decoded bits were compared to the transmitted bits to calculate the detection error.

As mentioned in Table 1, the maximum velocity was equal to $v_{\max}=0.9\;m/s$. The maximum value for the subcarrier frequencies for Config. #1 and Config. #2 were 130.5 kHz and 150 kHz, respectively. As such, using Equation (31) described in Section 3.2, the respective maximum Doppler shifts were equal to 78.3 Hz and 90 Hz. These frequency shifts are smaller than the maximum tolerable Doppler shift of either of the proposed systems, as calculated in Table 2. Therefore, the pilot placement should work efficiently in this scenario.

To improve the performance of the OFDM communication link subject to frequency selectivity, a simple 1/3 repetition code was applied. At the receiver side, repetition decoding was done using majority logic detection. Although this technique provides limited gain, the purpose was to demonstrate the reliability of the Doppler compensation method.

To predict the reliability of the proposed Doppler compensation method, a model of the communication link was developed. Initially, the model assumed that the time-varying delay of the strongest path is known and used for Doppler compensation. In a second model, the channel estimation algorithm was also included to accurately represent a fully automated receiver physical layer.

**Figure 9:**
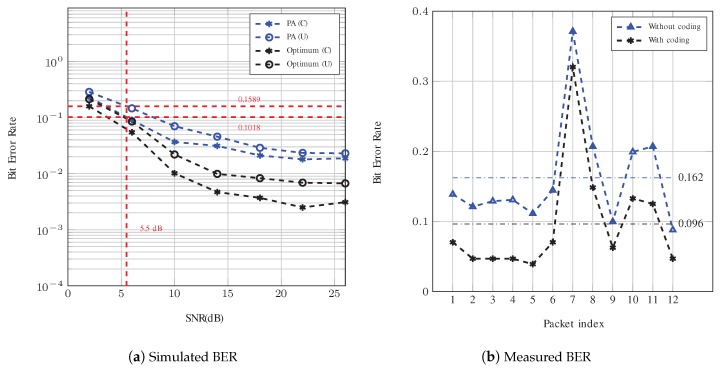
BER analysis of Config. #1. The red dotted lines in (**a**) show the value of BER at 5.5 dB for system with PA compensation (with and without coding). The horizontal lines in (**b**) are used to identify the average measured BER.

**Figure 10:**
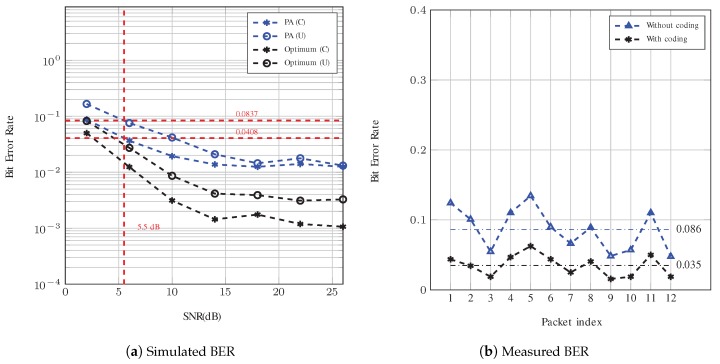
BER analysis of Config. #2. The red dotted lines in (**a**) show the value of BER at 5.5 dB for system with PA compensation (with and without coding). The horizontal lines in (**b**) are used to identify the average measured BER.

The simulated BER for Config. #1 and Config. #2 are shown in Figure 9a and Figure 10a, respectively. As observed, the proposed pilot aided (PA) algorithm shows a BER performance better than 0.05 at high SNR (higher than 15 dB). As expected, the optimum equalization generally has a much better BER performance than the pilot aided compensation scenario. In addition, a comparison between the BER curves for Config. #1 with 64 pilot sub-carriers and Config. #2 with 96 pilot sub-carriers shows that the Doppler and channel estimation was improved by increasing the number of pilot sub-carriers.

In the simulation, note that the channel model represents a multi-scale multipath scenario in which each path arrival has a different time-scaling factor. Therefore, the Doppler compensation with respect to the time-varying delay of the strongest path (optimum scenario) is not perfect in either of these systems. As a result, the BER curves reach an error floor because the inter-carrier interference cannot be entirely removed.

The BER of both systems was evaluated through measurements and the results are presented in Figure 9b and Figure 10b. For Config. #1, the bit rate is 560 bps and 1680 bps with and without coding, respectively. According to Figure 9a, the corresponding BERs for this configuration using PA compensation at SNR = 5.5 dB are simulated to be 0.102 and 0.159. These values are very close to the average measured BERs, which are 0.096 and 0.162, respectively, for a system with and without repetition coding. Note that the 95% confidence interval for the measured BERs of the coded and uncoded system are in the ranges of (0.153,0.172) and (0.082,0.112), respectively.

For Config. #2 with and without repetition coding, the bit rates are 776 bps and 2328 bps, respectively. As shown in Figure 10a, the simulated BERs at an SNR equal to 5.5 dB after Doppler compensation with and without coding are 0.041 and 0.084, respectively. According to Figure 10b, the measured respective BERs are 0.035 and 0.086, which are very close to the simulated values. More specifically, the BER was measured with a 95% confidence interval to be (0.029,0.041) and (0.082,0.091) with and without coding, respectively. The comparison between the measurements and simulation for Config. #2 are even closer in comparison to those of Config. #1. This is attributed to the fact that the channel conditions are more stable during this measurement.

### 4.2. Long-Range Reliable Link

The Doppler shift compensation algorithm was applied to a low-frequency narrowband system deployed over a range of 10 km. This link was intended for the exchange of low bitrate command messages at high reliability. As demonstrated, when the transmitting and receiving platforms were immobile, Doppler compensation was unnecessary, but the algorithm provided an improvement when the transmitter was moving.

In summer 2017, a three-day trial was run in the East Coast of Nova Scotia, near St-Margarets’s Bay. A receiver with a five-element vertical line array was moored at 35 m from the surface, in a water depth of 80 m for the duration of the experiments. A 2-kHz transmitter with a bandwidth of 300 Hz was deployed from a vessel. The vessel was anchored at pre-defined coordinates to characterize the performance at five different ranges from 1 km to 10 km. Two additional communication tests were run: in the first, the vessel was allowed to drift, and, in the second, the captain piloted the boat in small concentric 30-m radius circles at an approximate speed of 1 knot and at 4 km from the receiver. Relatively low-speed was maintained to protect the transmit apparatus, and avoid entanglement with the vessel motor.

**Figure 11:**
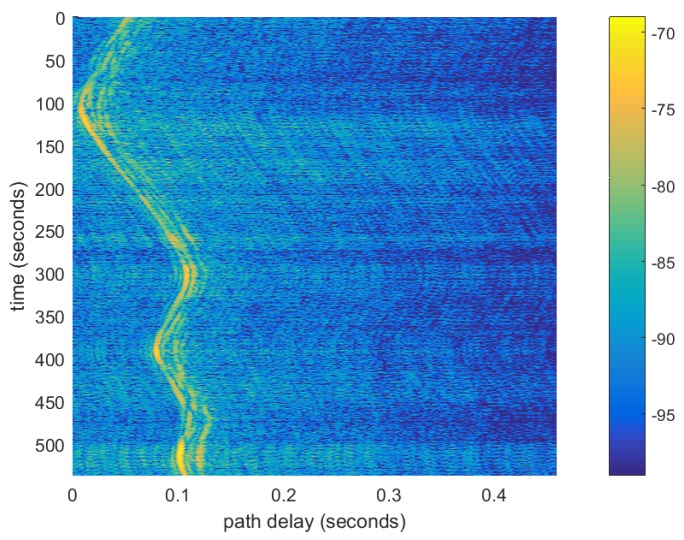
Channel impulse response as a function of time for the long range experiment.

The link channel impulse response is shown in Figure 11 measured using a set of concatenated pseudo-random sequences. As can be seen, over the 9-min period, there was significant Doppler scaling that induced a variable delay of arrival between the OFDM symbols.

The payload transmitted consisted of 12 consecutive frames. Each frame contained nine OFDM symbols with 512 subcarriers and a 400-chip long cyclic prefix. The first symbol was intended for synchronization and channel estimation. It contained 512 pilot sub-carriers. All other OFDM symbols had 256 null sub-carriers, 126 payload carriers, and 130 pilot sub-carriers. The total transmit window duration was 405 s.

The receiver recorded the information for the entire duration of the trials. Post-processing was used to characterize the acoustic propagation conditions as well as receiver performance. In the communication band, the SNR was high, above 30 dB for all test ranges. For the tests in which the transmitter vessel was carefully anchored, the Doppler spread was measured to be approximately 0.2 Hz, and as such the coherence time was on the same order of magnitude as the 2.1-s OFDM symbol duration (without cyclic prefix). In these conditions, the Doppler compensation technique described in Section 3.2 did not improve the reliability of the link, or only marginally.

For the mobile scenarios, the time-scaling factor ${\hat{\alpha }}_{m}$ was estimated at the *m*th OFDM symbol and was compensated. For compensation, the time-scaling factors ${\hat{\alpha }}_{m}$ for m=1,⋯,8 were interpolated such that an equivalent symbol arrival time $\tau_{rx}\left(t\right)$ was obtained at a discrete time interval sampled at a rate of 10.240 kHz. To rescale the received signal, the TVFDL filter used a correction delay $\tau_{corr}\left(t\right)$. The correction delay $\tau_{corr}\left(t\right)$ was fixed at $-\tau_{rx}\left(t\right)$, so that the corrected symbol arrival time became constant and equal to 0.

**Figure 12:**
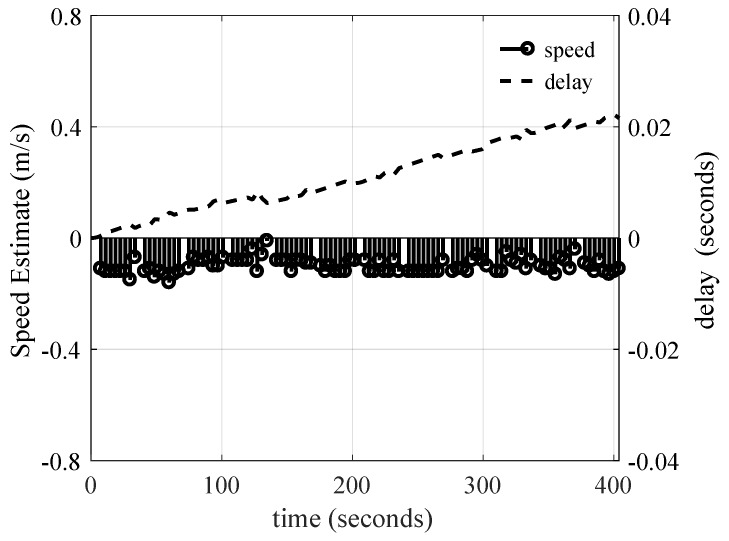
Output of the velocity estimation algorithm, and resulting symbol delay correction while the transmit vessel was drifting.

The time-scaling estimation algorithm was assessed when the transmit vessel was drifting. Note that, when this test was run, the transmitter was at 2 km from the receiver. As shown in Figure 12, the transmitter velocity was estimated in the direction of propagation. The compensation delay is also shown to rescale the signal. As can be observed, the equivalent velocity was generally constant at a value of −0.1 m/s. The negative sign indicates that the transmitter was moving towards the receiver. As can be seen, since the velocity was constant, the compensation delay increased, to a first-order approximation, linearly with time. At the end of the 400-s delay, the compensation was 24 ms.

**Figure 13:**
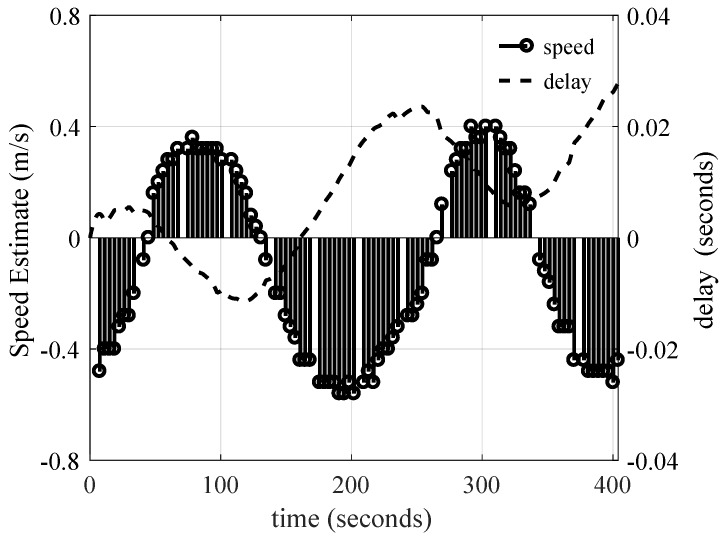
Output of the velocity estimation algorithm, and resulting symbol delay correction at the 4-km range. The transmit vessel was moving at approximately 1 knot in small concentric circles with a radius below 50 m such that the boat was moving towards the receiver for a period of time, and then away from the receiver.

Next, the time-scaling was estimated when the boat was moving in a circular motion. When this test was run, the distance between the transmitter and receiver was 4 km. As shown in Figure 13, the maximum absolute speed of 0.5 m/s (1 knot) was estimated, consistent with the physical boat speed that the captain monitored. Note that the speed estimation curve follows a sinusoidal-like shape since for some duration the transmitter was driving towards the receiver, and otherwise it was going away. The correction delay $\tau_{corr}\left(t\right)$ used to compensate for the boat mobility is also shown in Figure 13. As can be observed, when the boat was moving at a negative velocity, the compensation delay increased, otherwise it decreased.

The communication link reliability was also simulated for the long range application. The frequency domain equalizer implemented in Section 4.1 was also used, and, to improve performance, the outputs of each OFDM symbol were combined to implement repetition coding. Since each frame contained eight OFDM symbols with payload, the performance was tested for repetition rates $R_{c}$ of 1, 2, 4 and 8. The useful bit rate was calculated accordingly. Maximum ratio combining (MRC) was used to weight the output of the five receiver elements. The MRC filter coefficients were obtained from the interpolated channel estimated using each OFDM symbol’s pilot tones.

**Figure 14:**
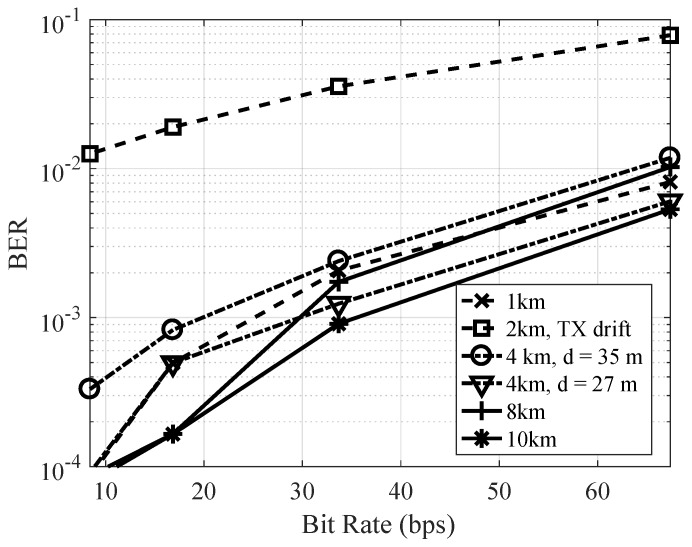
Measured bit error rate without Doppler compensation as a function of data rate for different ranges.

The bit error rate of the OFDM symbol without Doppler compensation is shown in Figure 14 as a function of bit rate for the different transmitter deployment stations. Note that the mobile deployment when the engines were turned on is omitted from this figure. The performance for the 2 km station was clearly much poorer than for the other station because the boat was drifting. Otherwise, for all other stations, the performance was relatively good and, for a bit rate of 16 bps, the probability of bit error was below $10^{-3}$.

**Figure 15:**
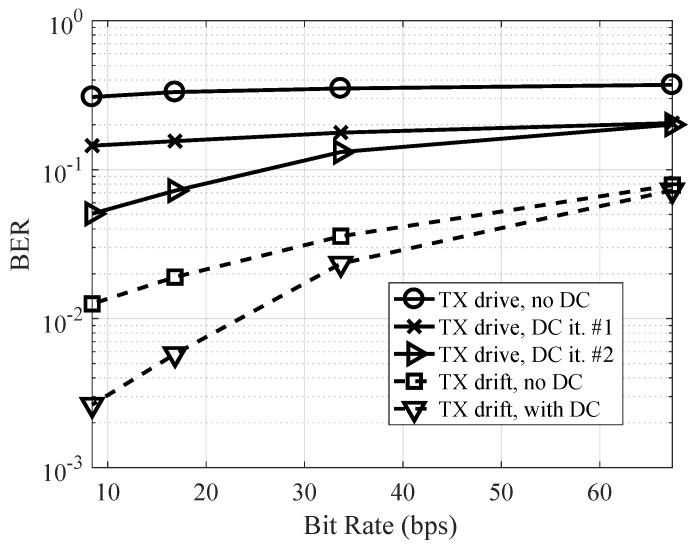
Measured bit error rate with Doppler compensation as a function of data rate for mobile conditions.

Next, the bit error rate of the OFDM symbol with Doppler compensation is shown in Figure 15 as a function of useful bit rate for the mobile conditions. As can be observed, the OFDM performance was very poor without compensation, particularly for the test in which the transmitter vessel engines were turned on. In this scenario, after one Doppler compensation iteration, the performance was only improved marginally. This is attributed to interpolation errors when the time-scaling factor varies significantly during an OFDM symbol. Consequently, two Doppler estimation and compensation iterations were run to improve the performance. After two iterations, improvements were no longer observed, and, for a bit rate of 8 bps, the BER was improved to 0.05 after two iterations for the scenario in which the vessel’s engines were turned on. Although the performance was improved, this highlights the difficulty of establishing a reliable link using OFDM in mobile conditions. When the boat was drifting, the original Doppler shift was more benign, and the output BER was 0.003 for a bit rate of 8 bps. More efficient coding techniques can be applied to further improve the performance in mobile conditions.

## 5. Conclusions

In this work, a Doppler compensation technique was developed, modelled and tested in realistic environments. Doppler shift is included in the channel model by introducing a time-variant time-scaling factor. A low-complexity implementation of the model that uses a time-variant fractional delay line (TVFDL) custom filter realization is also described.

At the OFDM receiver, estimation and tracking of the filter is realized by minimizing the error between the OFDM symbol and its estimate. To improve communication reliability, the Doppler effect during the transmission of a frame is corrected by interpolating the time-scaling estimate between the symbols.

While the TVFDL filter developed in this work can realize real-time Doppler compensation, an estimate of the Doppler scaling factor at the OFDM receiver can only be produced at the OFDM symbol rate, and as such it is required to buffer the content of consecutive symbols to interpolate the time-scaling factor between OFDM symbols. Therefore, an algorithm that can estimate the Doppler scaling factor in the time domain is highly desirable to maintain real-time operation.

The algorithm was tested in realistic conditions for two extreme applications and the output of the simulator was compared to that of the measured results. The channel prediction and BER estimator provided by the model were in close agreement with the measured results. For a short range telemetry application, a 2.4 kbps link was established, and a bit error rate of 0.03 was measured at 5.5-dB SNR. In addition, a 16-bps link was measured for a distance as long as 10 km. For fixed platforms, the BER was below $10^{-3}$. When the receiver was mobile, BER improvements were demonstrated when the Doppler correction filter was enabled. For example, when the receiver was moving at 1 knot, two iterations of the Doppler compensation filter improved the bit error rate from 0.2 to 0.06. It can be expected that, for both applications, an efficient error correcting coding technique across the subcarriers will provide significant performance improvement.

## Figures and Tables

**Table 1:** Sea trial environmental characteristics.

Parameter	Value
Total depth	28 m
Depth of the transmitter	26.5 m
Depth of the receiver	1.5 m
Initial distance ($d_{0}$)	68 m
Initial velocity ($v_{0}$)	−0.29 m/s
Maximum velocity ($v_{\max}$)	−0.9 m/s
Average acceleration (*a*)	−0.001 $m/s^{2}$
Signal to noise ratio	5.5 dB

**Table 2:** OFDM parameters for the two short-range configurations.

Parameter	Config. #1	Config. #2
Number of subcarriers (*N*)	512	1024
Data subcarriers ($K_{d}$)	64	160
Pilot subcarriers ($K_{p}$)	64	96
Null subcarriers ($K_{n}$)	384	768
Data subcarriers per frame ($P_{d}$)	2	4
Pilot subcarriers per frame ($P_{p}$)	2	3
Null subcarriers per frame ($P_{n}$)	6	12
Bandwidth (*B*)	19 kHz	20 kHz
Carrier frequency ($f_{c}$)	121 kHz	130 kHz
Fractional bandwidth $\left(B/f_{c}\right)$	15.7%	15.4%
Frequency separation (Δf)	37.1 Hz	19.5 Hz
Max. tolerable freq. shift ($P_{n}\cdot \Delta f$)	222.6 Hz	234.4 Hz
Block duration ($T_{b}$)	33.6 ms	60.2 ms
Cyclic prefix duration ($T_{cp}$)	6.4 ms	9 ms
Symbols/packet (*M*)	6	6
Length of preambles	27.2 ms	51.2 ms
